# Supplementary material for: Hydration Fingerprints: A Reproducible Protocol for Accurate Water Uptake in Anion-Exchange Membranes
Source: Membranes (Basel). 2025 Aug 28;15(9):257. doi: 10.3390/membranes15090257 (PMC12472172; doi:10.3390/membranes15090257)
Supplement: Supplementary file 1 [file membranes-15-00257-s001.zip › membranes-3789769-supplementary.pdf]

Supplementary

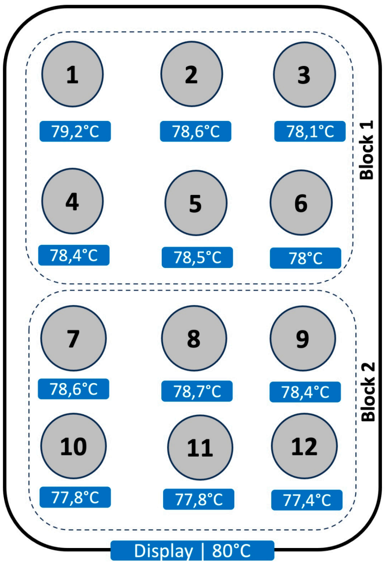

Figure S1. Mapping of temperature uniformity across the 12-position heating block. Each numbered position corresponds to a sample well on the heating plate set to 80 °C (display). Measured temperatures at all positions fall within ±1.2 °C of the set point, confirming homogeneous thermal distribution across both blocks.

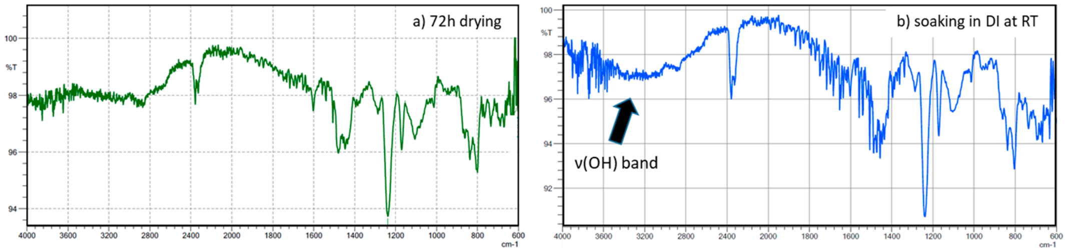

Figure S2. ATR-FTIR spectra of FAAM-PK-75 during drying and after soaking in deionized water. (a) After drying over silica gel, a residual ν(OH) band at ~3405 cm^{-1} remains, indicating incomplete dehydration. (b) Following 24 h soaking in deionized water (and blotting via standardized method).

Table S1. ATR-FTIR vibrational band assignments for FAAM-PK-75. Key peaks are listed with their approximate wavenumbers and corresponding functional-group or polymer backbone vibrations, including the water ν(OH) stretch at ≈3405 cm^{-1} [9, 11, 14, 22].

Wavenumber / cm^{-1}	Assignment (PK-75)
≈ 3405	ν(OH) stretch of absorbed water
3050–3010	Aromatic C–H stretch (polysulfone backbone)
2960–2850	Aliphatic C–H stretch (quaternary ammonium CH_3)
1640	Bending of water
1590 ± 10	Aromatic C=C stretch (polysulfone); overlaps reported 1580 cm^{-1} 'water dimer' band
1480–1450	CH_3 asymmetric bend ($N^+(CH_3)_3$)
1320–1290	SO_2 symmetric stretch (polysulfone)
1170–1150	SO_2 asymmetric stretch (polysulfone)
1040–960	$C–N^+$ stretch of quaternary ammonium
830–810	Aromatic C–H out-of-plane bend

625 ± 5	C–SO_2 torsional vibration

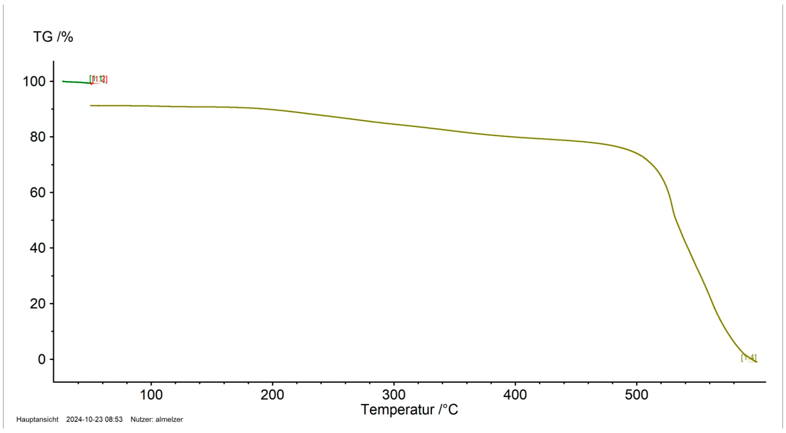

Figure S3. Thermogravimetric analysis (TGA) of FAAM-PK-75. The sample (~8.75 mg) was heated from 25 °C to 600 °C at 10 °C min^{-1} under a $N_2/O_2/N_2$ atmosphere (20/20/20 mL min^{-1}) in an Al_2O_3 crucible. Less than 1 % mass loss is observed up to 200 °C, confirming the membrane's thermal stability well above typical operating and soaking conditions. A gradual mass decrease beyond 200 °C reflects polymer backbone decomposition, with major degradation occurring above ~350 °C.

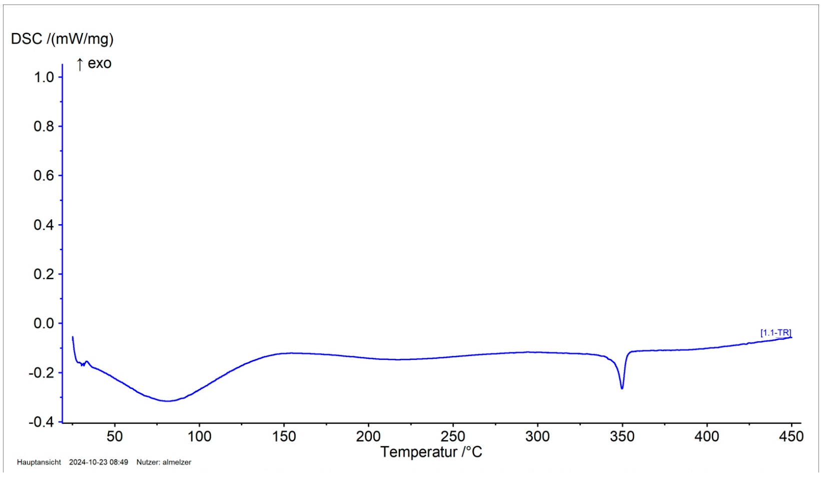

Figure S4. Differential scanning calorimetry (DSC) of FAAM-PK-75. A ~10 mg sample was scanned from 25 °C to 450 °C at 10 °C min^{-1} under N_2 purge (40 mL min^{-1}) using a concave aluminum pan with pierced lid. A weak, broad endothermic feature appears below ≈150 °C, indicative of minor segmental relaxation—while no sharp melting or crystallization events are observed. A more pronounced endotherm starts above ≈350 °C, corresponding to the onset of polymer backbone degradation.

Table S2. Comparison of Chain Length, Steric Bulk, Relativ polarity, Dielectric Constant, and Viscosity [29–32].

Solvent	Chain Length	Steric Bulk (qualitative)	Relative polarity	Dielectric Constant (ε, 25 °C)	Viscosity (cP, 25 °C)
Water	0	1,0	High	78.5	1.00
Ethanol (EtOH)	C_2H_5OH	Low (linear)	0,654	24.3	1.08
Isopropanol	$(CH_3)_2CHOH$	Moderate (branched)	0,617	18,3	2.038

Glycerol	$C_3H_8O_3$	High (multiple –OH)	0,812	42.5	945
Solvent	Chain Length	Steric Bulk (qualitative)	Relative polarity	Dielectric Constant (ε, 25 °C)	Viscosity (cP, 25 °C)

Table S3. Manufacturer-reported water uptake and dimensional swelling for commercial Fumatech AEMs under specified conditioning. Counter-ion form, soaking medium, and test conditions are from Fumatech technical specifications; uptake and swelling values reflect gravimetric and dimensional changes after 24 h immersion [10]

Membrane	Counterion/ Form	Soaking Medium & Conditions	Reported Uptake/Swelling (Fumatech)	Source
FAA-3-20	Br^- form	DI H_2O, 25 °C, 24 h (Br^- soak)	Water uptake ≈ 7 wt %; swelling < 2 % (length or thickness)	Fumatech FAA-3-20 Specification PDF
FAA-3-50	Br^- form	DI H_2O, 25 °C, 24 h (Br^- soak)	Water uptake ≈ 25 wt %; thickness increase ≈ 15–20 %	Fumatech FAA-3-50 Specification PDF
FAAM-PK-75	OH^- form (after KOH soak)	9 M KOH, 20 °C, 24 h ($Br^- \rightarrow OH^-$ exchange)	Water content ≈ 40–60 wt %; thickness increase ≈ 20–40 %	Fumatech FAAM-PK-75 Spec. Sheet

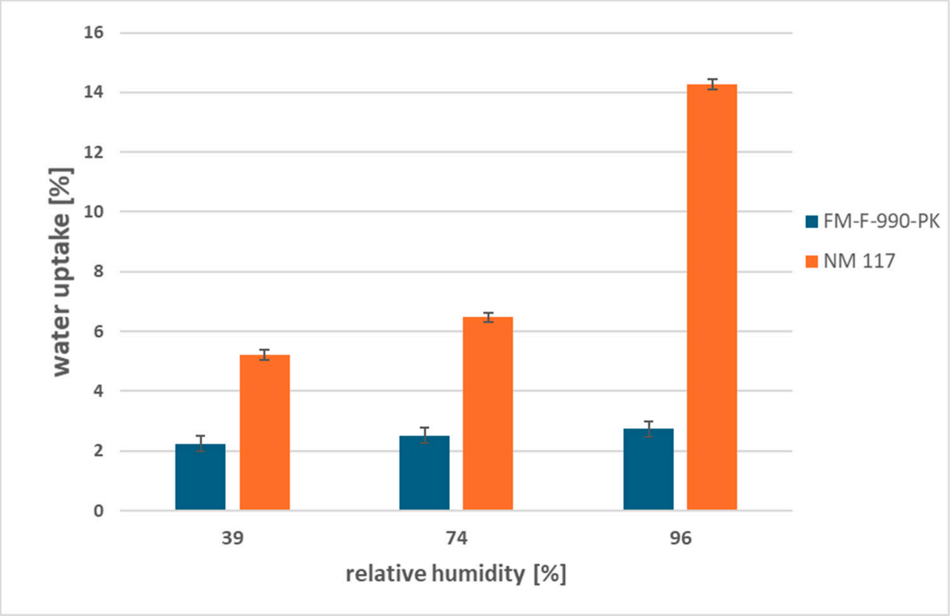

Figure S5. Vapor-phase water uptake of (a) the PEEK-reinforced PEM FM-F-990-PK and (b) Nafion™ N117, measured under the same RH conditions (≈ 39 %, 74 %, 96 % at 23 °C) and blotting protocol. The reinforced FM-F-990-PK plateaus at ~2 wt %, whereas unreinforced Nafion™ N117 swells progressively from ~5 wt % to ~14 wt %, demonstrating the method's sensitivity to membrane architecture.
